# Trends in noninvasive ocular nanoparticle drug delivery: A bibliometric analysis (2004–2023)

**DOI:** 10.17305/bb.2025.11772

**Published:** 2025-03-04

**Authors:** Dan Li, Qing Ye, Chao Li

**Affiliations:** 1 Suzhou Kowloon Hospital, Shanghai Jiaotong University School of Medcine, Jiangsu Province, China

**Keywords:** Nanoparticle, drug delivery systems, transocular surface, bibliometric, visualization analysis

## Abstract

This study presents a bibliometric analysis of research on noninvasive nanoparticle drug delivery systems for the transocular surface from 2004 to 2023. Relevant publications were retrieved from the Web of Science Core Collection. VOSviewer and CiteSpace were used to map contributions by countries/regions, authors, institutions, journals, keywords, keyword clusters, and timeline trends. A total of 695 articles were analyzed, showing a steady year-by-year increase in publications. China, the United States, and Spain were the leading contributors. Among authors, Alvarez-Lorenzo, Carmen was the most prolific, while Chanhan, Anuj’s work received the most citations among the top 10 prolific researchers. The International Journal of Pharmaceutics published the highest number of articles in this field, whereas the Journal of Controlled Release was the most frequently cited among the top 10 most productive journals. The University of Santiago de Compostela and the University of Florida were among the most active institutions in this research area. Keyword analysis identified recent key themes, such as controlled release, cell interaction, dry eye, mechanisms, gene expression, and ocular drug delivery. The growing interest in transocular surface nanoparticle drugs is driven by their advantages, including increased solubility, improved stability, reduced administration frequency, sustained therapeutic concentrations, enhanced corneal penetration, and prolonged ocular surface residence time.

## Introduction

The global ocular drug delivery systems market continues to expand. Between 2017 and 2021, this market maintained a steady compound annual growth rate (CAGR), and this upward trend is expected to persist, with significant market expansion projected by 2028. Topical medication remains the primary treatment approach for various ocular diseases. However, traditional drug delivery methods—such as eye drops, ointments, and tablets—face significant limitations, including short drug retention times due to rapid tear drainage, limited permeability, patient adherence challenges, and potential side effects from frequent dosing [1–3]. As a result, these conventional methods often fail to provide long-term, effective, and patient-friendly treatment solutions for ocular surface diseases, including dry eye, infections, glaucoma, myopia, and fundus disease. A key innovation in ophthalmic diagnosis and treatment is the development of noninvasive transocular drug delivery systems, which have demonstrated significant practical value and technological advancements in overcoming the unique challenges of ocular diseases. These advancements are driven by rapid progress in biomaterial science, drug formulation technology, and nanotechnology. Notably, these new delivery systems address major anatomical barriers, such as the corneal barrier, tear washout, and the complex physiological environment of the ocular surface, which traditionally hinder drug absorption and sustained efficacy [4–6]. Noninvasive transocular drug delivery systems offer several advantages, including the ability to bypass anatomical barriers, achieve precise targeting and controlled release, enhance bioavailability, and minimize drug loss in systemic circulation. The integration of novel nanomaterials has further accelerated progress in this field. Due to their unique size effects, surface properties, and drug-loading capacities, these nanomaterials enable more precise drug targeting, prolonged therapeutic effects, and noninvasive administration to the ocular surface. By analyzing the design principles, material choices, and real-world applications of these advanced systems, we can better understand their significant contributions to ophthalmic medical technology [2, 7–9]. Bibliometrics—a field that applies mathematical and statistical methods to analyze scientific literature—originated in the 1950s through the pioneering work of Eugene Garfield, who laid the foundation for quantitative studies of scientific publications [10]. Since then, bibliometric analysis has provided valuable insights into the growth patterns of scientific knowledge, collaboration networks, and citation dynamics. Today, it is widely used to evaluate research impact, calculate journal impact factors, analyze discipline structures, and map research collaboration networks, offering objective and quantitative support for research management and decision making [11–14]. To date, no comprehensive bibliometric review has examined the research status and evolving trends in transocular surface nanoparticle drug delivery systems. The core aim of this study is to systematically analyze and summarize research on noninvasive ocular surface nanoparticle drug delivery systems from 2004 to 2023 using bibliometric methods. We focus on this field not only because of its significant potential and applications in ophthalmic therapy but also due to the many challenges and uncertainties it presents. By employing bibliometric techniques, this study quantitatively maps the field’s developmental trajectory, research hotspots, and evolutionary trends over the past two decades. Visualization diagrams further illustrate the field’s overall framework, highlighting recent research focal points and emerging trends.

## Materials and methods

### Data collection

**Figure 1.**
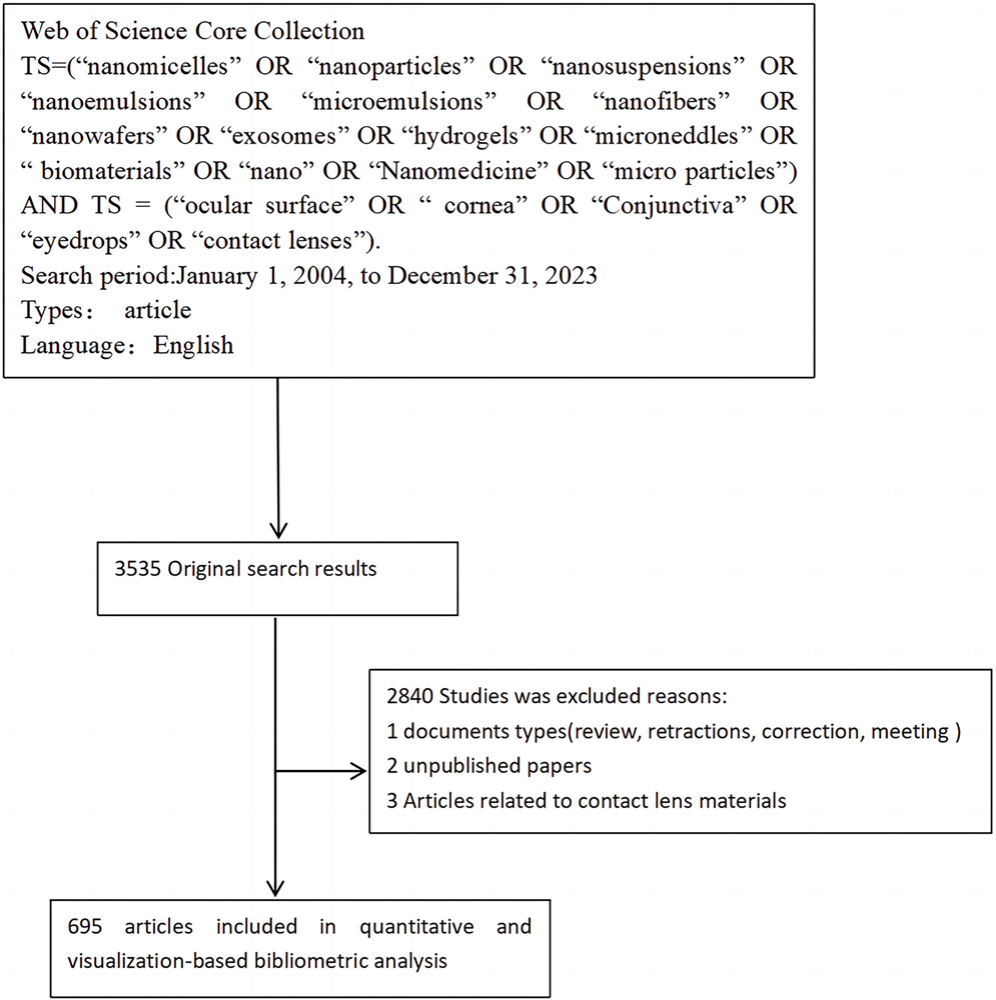
The data collection and retrieval strategy.

The Web of Science Core Collection (WoSCC) was used as the data source for this study. Since it covers a wide range of subject areas, it ensures a comprehensive literature search on noninvasive nanoparticle transocular drug delivery systems. This database rigorously screens and reviews publications to maintain the accuracy and credibility of search results. To obtain comprehensive and precise data, we selected the SCI-EXPANDED and SSCI citation indexes. After the initial literature collection, we used software such as EndNote to remove duplicate bibliographic records. By comparing key information—such as titles, authors, and abstracts—we ensured the uniqueness of each record, preventing duplicate counts from affecting the study results. The search terms used were: TS ═ (“nanomicelles” OR “nanoparticles” OR “nanosuspensions” OR “nanoemulsions” OR “microemulsions” OR “nanofibers” OR “nanowafers” OR “exosomes” OR “hydrogels” OR “microneedles” OR “biomaterials” OR “nano” OR “nanomedicine” OR “microparticles”) AND TS ═ (“ocular surface” OR “cornea” OR “conjunctiva” OR “eye drops” OR “contact lenses”). The search timeframe spanned from January 1, 2004, to December 31, 2023. Only articles written in English were included. After removing duplicates, a total of 695 valid papers were retrieved. The screening process is illustrated in Figure 1.

### Methods

In this study, we employed bibliometric methods with the assistance of two visualization tools: VOSviewer and CiteSpace. Specifically, we used VOSviewer (1.6.20) and CiteSpace (6.1) to generate knowledge graphs. Each software has its own strengths and can complement the other. CiteSpace utilizes a set-theoretic data standardization method to measure the similarity of knowledge units. Its similarity algorithm generates Timezone and Timeline views within a time slice, providing a clear representation of knowledge evolution and the historical span of specific literature clusters. This helps illustrate the development process and emerging trends in the field [15–17]. VOSviewer, on the other hand, applies a probability-based data standardization method and offers multiple visual perspectives, including keyword co-occurrence, co-authorship networks, and thematic mapping. Its network, overlay, and density views are particularly known for their user-friendly interface and visually appealing graphics [18, 19].

## Results

### Overall research publication information

**Figure 2.**
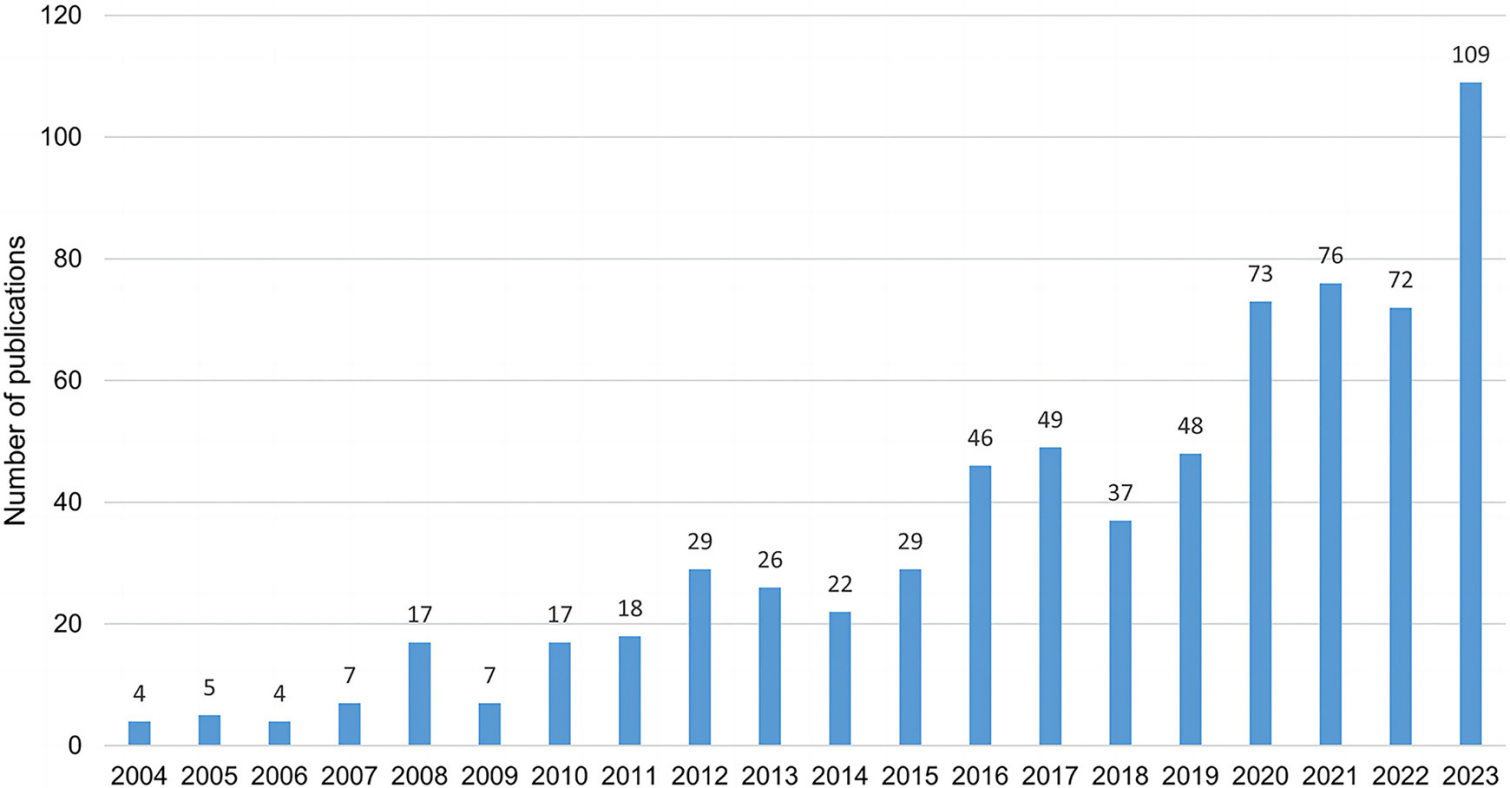
Distribution of publications from 2004 to 2023.

The number of publications and their trends over the years reflect the overall significance and level of attention in the field. Visualizing the publication dates of research on noninvasive nanoparticle drug delivery systems provides insight into the research landscape, including its temporal distribution, annual publication volume, and development trajectory, as shown in Figure 2. The search results indicate that these 695 papers originate from 58 countries, involve 916 institutions, and include 3405 authors. They are published across 198 journals and cite 20,760 sources. From 2004 to 2023, the total number of publications has followed a generally increasing trend, despite some fluctuations. Before 2012, the annual publication volume remained consistently below 20 papers. Afterward, it fluctuated but never dropped below 20, peaking at 109 papers in 2023. As illustrated in Figure 2, research activity in this field has been growing steadily, reflecting a rising level of interest and engagement from researchers worldwide.

### Co-authorship analysis of authors, countries, institutions, and journals

**Figure 3.**
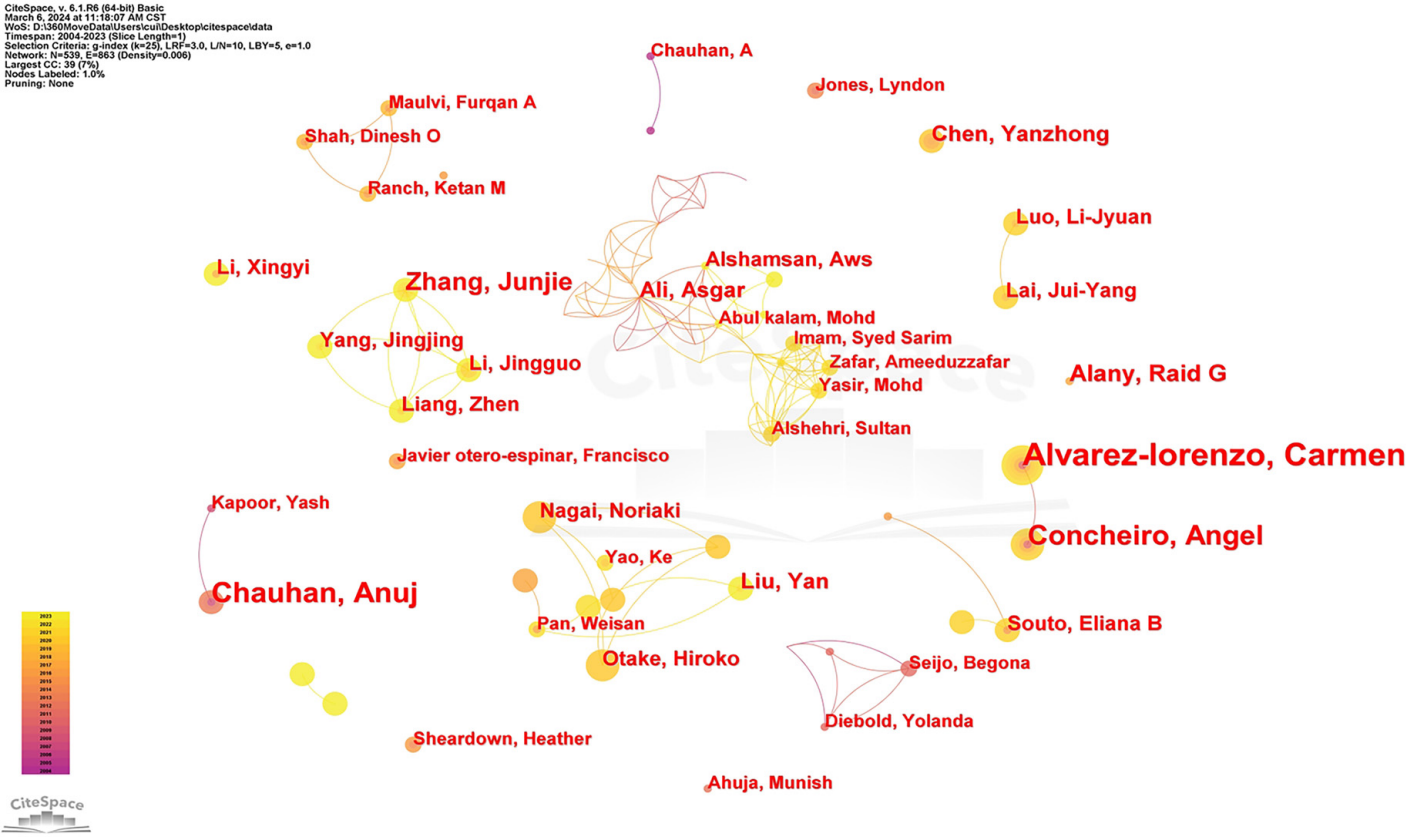
The co-authorship network of authors.

The analysis of literature authors provides insights into key scholars and core research forces in the field. Authors with more than three publications are classified as core authors, totaling 76, with a combined output of 322 papers—accounting for 46.33% of total publications. This aligns with Price’s half-standard, indicating a relatively stable network of author collaborations in the field. Using CiteSpace software, co-authorship networks in research on nanomediated ocular drug delivery systems were mapped (Figure 3). In this visualization, nodes represent authors, with node size positively correlated with publication count. Connections indicate collaborations, with link colors reflecting publication years. Different node colors represent papers published in different years, while nodes with multiple concentric rings indicate publications across multiple years. Figure 3 highlights influential authors and the intensity of their collaborations, helping researchers understand academic networks and research communities. Additionally, using VOSviewer, an analysis of high-productivity authors (those with more than 10 publications) is presented in Table 1. Among them, the most prolific is Alvarez-Lorenzo from Spain, with 16 publications, 475 citations, and an average of 29.69 citations per paper. Alvarez-Lorenzo’s work has significantly advanced nanoparticle-based ocular drug delivery and provided insights applicable to drug delivery in other organs. Through in-depth analysis of methodology, data, and clinical applications, we recognize that optimizing drug delivery systems is crucial for efficient drug administration. These findings offer valuable guidance for our research and future directions. Notably, the second most prolific author is Anuj Chauhan from the United States, with 14 publications, 1274 citations, and an impressive average of 92 citations per paper. In third place is Ángel Concheiro from Spain, with 13 publications, 384 citations, and an average of 29.54 citations per paper.

**Table 1:** The top 10 productive authors

**Rank**	**Author**	**Documents**	**Citations**	**Average citation/publication**
1	Alvarez-Lorenzo, Carmen	16	475	29.69
2	Chauhan, Anuj	14	1274	91.00
3	Concheiro, Angel	13	384	29.54
4	Zhang, Junjie	10	133	13.30
5	Majumdar, Soumyajit	9	463	51.44
6	Nagai, Noriaki	9	195	21.67
7	Sheardown, Heather	9	167	18.56
8	Li, Xingyi	8	180	22.50
9	Ali, Asgar	7	467	66.71
10	Lai, Jui-Yang	7	465	66.43

**Table 2:** The top 10 productive countries

**Rank**	**Country**	**Documents**	**Citations**	**Average citation/publication**
1	China	220	5248	23.85
2	USA	112	5279	47.13
3	India	80	3078	38.48
4	Spain	65	3182	48.95
5	Canada	39	1423	36.49
6	Egypt	35	737	21.06
7	Italy	33	732	22.18
8	Portugal	30	963	32.10
9	Saudi Arabia	29	740	25.52
10	Iran	29	485	16.72

**Figure 4.**
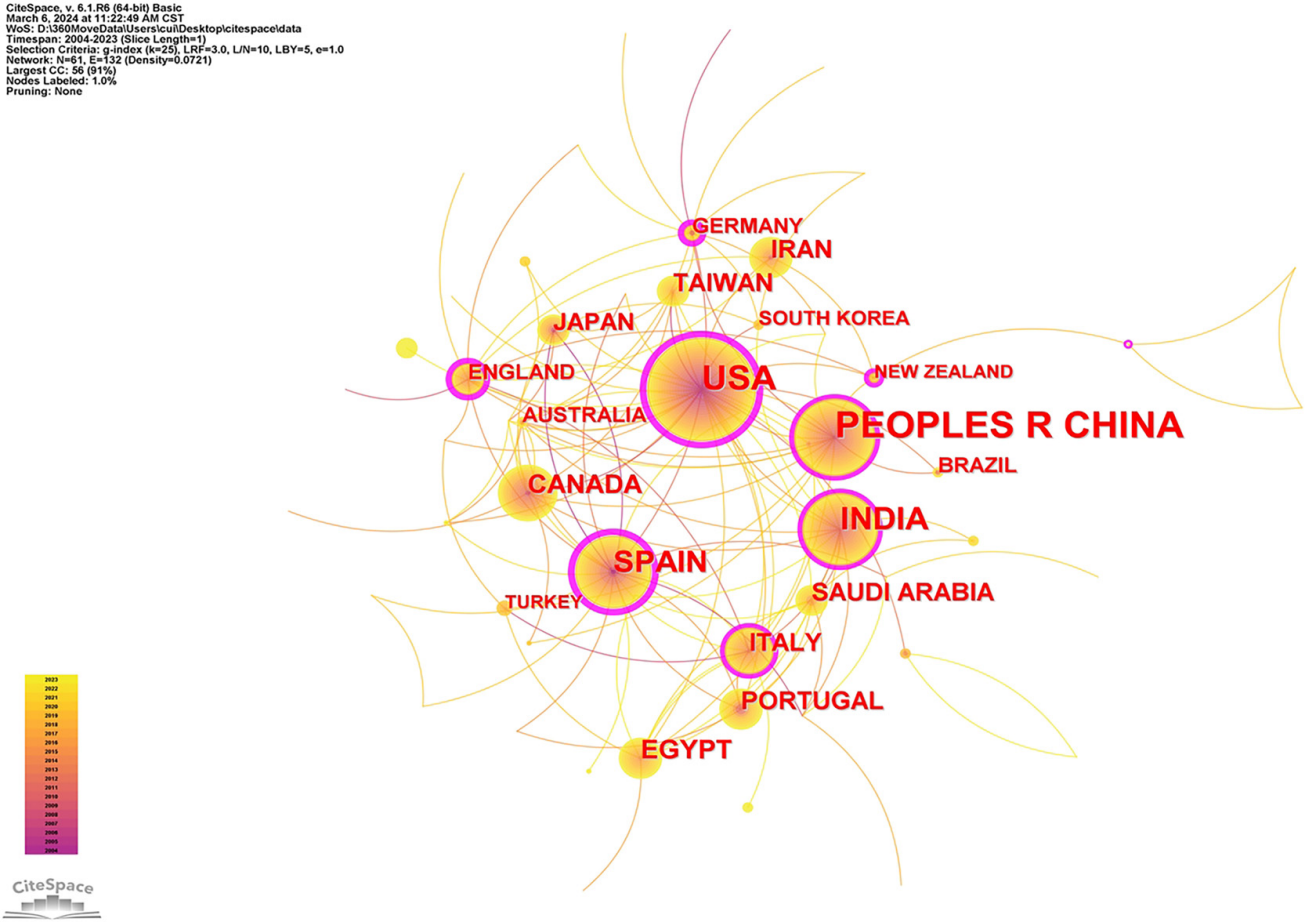
The co-authorship network of countries.

**Figure 5.**
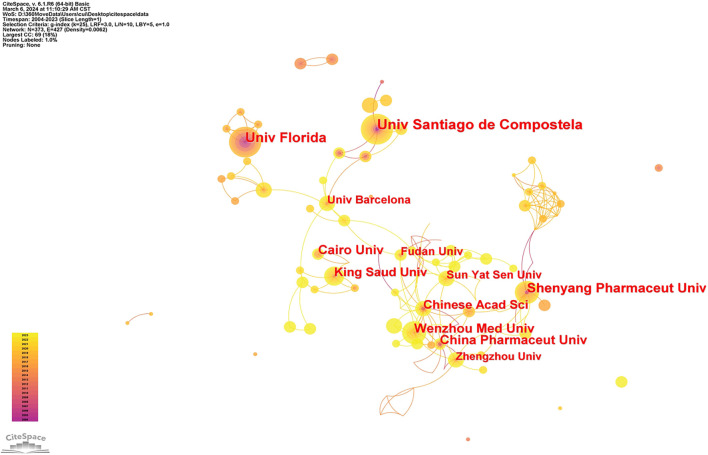
The co-authorship network of productive institutions.

To evaluate the most significant contributors in this research field, a visual analysis of publication volume by country was conducted. Initially, countries with at least 10 publications were analyzed using CiteSpace. The results, shown in Figure 4, indicate that larger nodes represent higher publication volumes, connections between nodes reflect collaboration strength (with thicker lines denoting more frequent collaborations), and node colors indicate publication time. Additionally, Table 2 (generated using VOSviewer) lists the top 10 countries based on publication volume and their average citation counts. Among the four countries with more than 50 cumulative publications—China (*n* ═ 220), the USA (*n* ═ 112), India (*n* ═ 80), and Spain (*n* ═ 65)—China leads with the highest research output in nanomediated ocular drug delivery systems. Countries with a purple outer ring in the graph exhibit high centrality, indicating their strong influence in the field. Notably, the four countries with the highest publication volumes also display high centrality, establishing them as core research forces. Furthermore, countries like England, Germany, and New Zealand, despite having lower publication volumes, exhibit high centrality, underscoring the impact of their research. Research institutes in these countries have formed stable collaborative networks. The United States has a strong track record of innovation in ocular drug delivery systems and maintains close international collaborations. Indian institutions have made notable progress in nanotechnology applications for ocular disease treatment, particularly in drug delivery. Spanish institutions contribute a unique perspective to nanoparticle design and drug delivery mechanisms. To further analyze the key research institutions, CiteSpace was used to examine the sources of literature on noninvasive nanoparticle drug delivery systems. The co-occurrence network diagram in Figure 5 displays institutions with at least 10 publications. Larger nodes indicate higher research output, while connection strength and thickness represent collaboration intensity. Node color signifies publication time, providing insights into institutional research impact. Additionally, Table 3 (analyzed using VOSviewer) lists the top 10 publishing institutions and their average citation counts. The University of Santiago de Compostela leads with 29centerpublications and an average citation count of 64.10, with its top publishing author, Alvarez-Lorenzo, affiliated with the institution. The University of Florida ranks second, with 22 publications and the highest average citation count (93.88), home to author Chanhan, Anuj. Shenyang Pharmaceutical University in China ranks third, with 20 publications, 545 total citations, and an average of 27.25 citations per paper.

**Table 3:** The top 10 productive organizations

**Rank**	**Organization**	**Documents**	**Citations**	**Average citation/publication**
1	UNIV SANTIAGO DE COMPOSTE LA	29	1859	64.10
2	UNIV FLORIDA	24	2253	93.88
3	SHENYANG PHARMACE UT UNIV	20	545	27.25
4	WENZHOU MED UNIV	18	204	11.33
5	CHINA PHARMACE UT UNIV	17	775	45.59
6	KING SAUD UNIV	16	443	27.69
7	CAIRO UNIV	16	394	24.63
8	UNIV BARCELONA	15	462	30.80
9	CHINESE ACAD SCI	14	559	39.93
10	SUN YAT SEN UNIV	14	378	27.00

**Figure 6.**
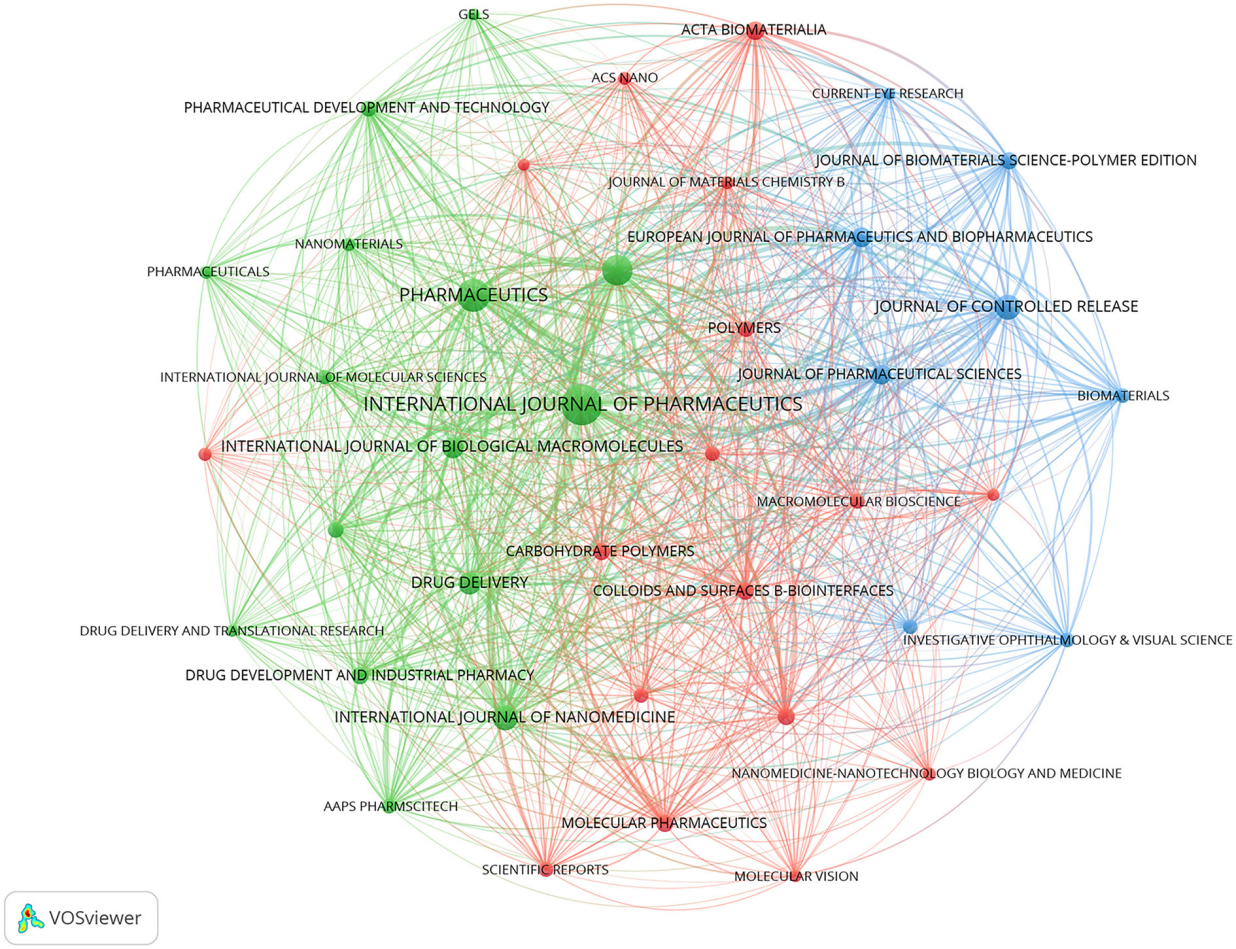
The co-authorship network of the journals.

VOSviewer software was used to analyze journals publishing research on nanomediated ocular drug delivery systems. A visual analysis of these journals was conducted, as shown in Figure 6. The results indicate that larger nodes represent journals with greater influence, while nodes of the same color reflect similarities in research areas. Table 4 highlights the top ten journals in this field. Journals with a publication count of 15 or more include International Journal of Pharmaceutics, Pharmaceutics, Journal of Drug Delivery Science and Technology, International Journal of Nanomedicine, Journal of Controlled Release, Drug Delivery, and International Journal of Biological Macromolecules. This suggests that these journals have played a significant role in advancing research in this area in recent years. An analysis of research papers published in these core journals reveals a focus on gene therapy, cell therapy, and biomaterials for treating ocular diseases. This trend underscores how advancements in biotechnology and nanotechnology are driving the emergence of new drug delivery strategies, offering fresh research ideas and directions for scientists in the field.

**Table 4:** The top 10 productive journals

**Rank**	**Organization**	**Documents**	**Citations**	**Average citation/publication**
1	INTERNATIONAL JOURNAL OF PHARMACEUTICS	57	2563	44.96
2	PHARMACEUTICS	36	497	13.81
3	JOURNAL OF DRUG DELIVERY SCIENCE AND TECHNOLOGY	30	395	13.17
4	INTERNATIONAL JOURNAL OF NANOMEDICIN E	22	814	37.00
5	JOURNAL OF CONTROLLED RELEASE	21	1290	61.43
6	DRUG DELIVERY	17	394	23.18
7	INTERNATIONAL JOURNAL OF BIOLOGICAL MACROMOLECULES	16	831	51.94
8	JOURNAL OF PHARMACEUTICAL SCIENCES	14	634	45.29
9	EUROPEAN JOURNAL OF PHARMACEUTICS AND BIOPHARMACEUTICS	13	964	74.15
10	ACTA BIOMATERIALIA	12	394	32.83

### Co-occurrence analysis of keywords

**Figure 7.**
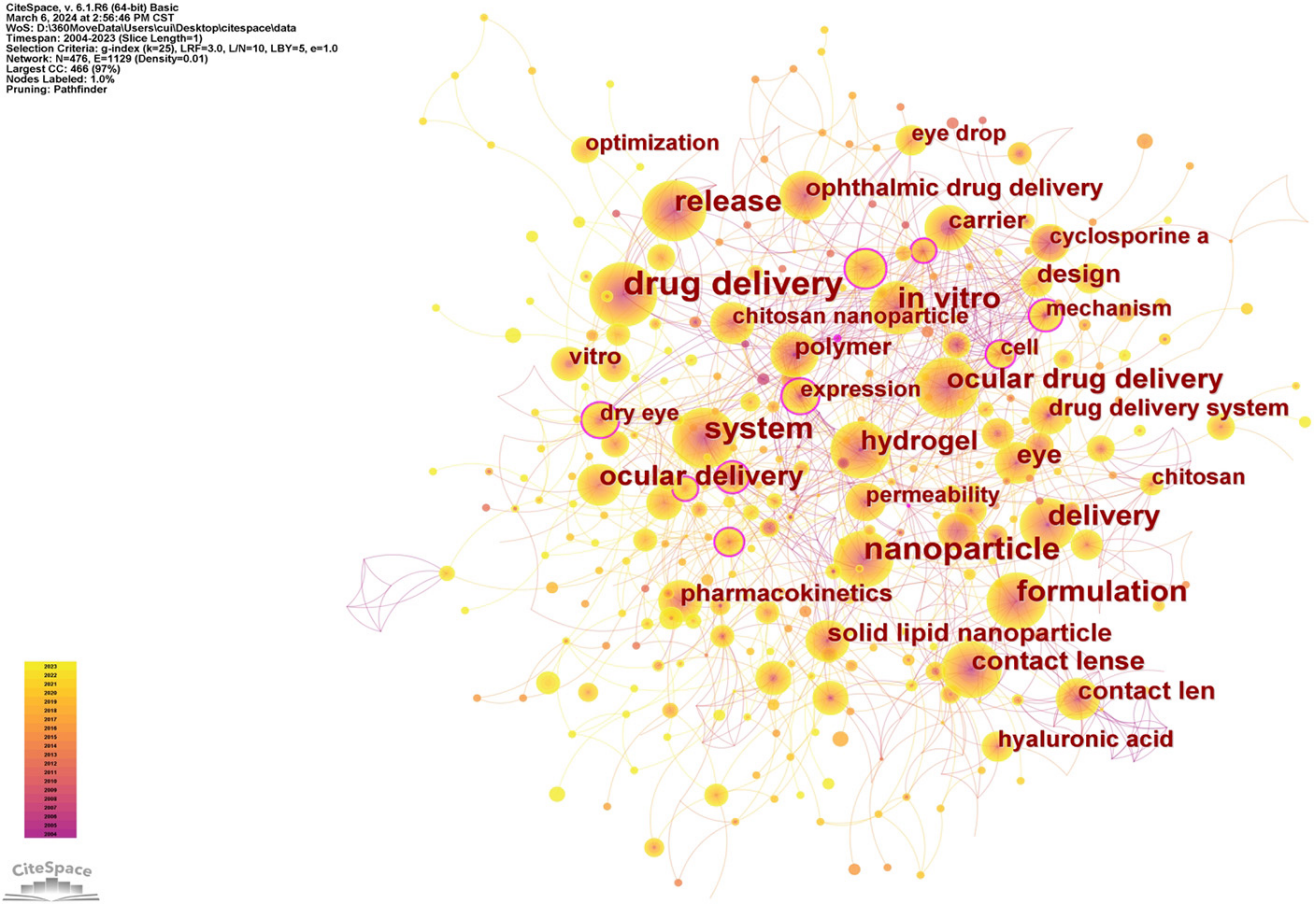
The co-occurrence analysis of keywords.

Keywords serve as a concise representation of a document’s content, and analyzing their co-occurrence can help identify research hotspots in a specific field. Using CiteSpace software, a co-occurrence network of keywords was constructed, as shown in Figure 7. In this network, nodes represent keywords, with their size and label font positively correlated with keyword frequency. Links indicate co-occurring keywords within the same article, with link colors corresponding to publication years on the left. Different node colors represent the publication years of the literature containing that keyword, while nodes with multiple concentric rings indicate publications spanning multiple years. The graph highlights keywords that appear at least 30 times, revealing 32 such keywords, including drug delivery, nanoparticle, system, release, *in vitro*, formulation, hydrogel, contact lens, and solid lipid nanoparticle (LNP), among others. Betweenness centrality measures a keyword’s importance within the network by assessing how often it acts as a bridge in the shortest paths between other nodes. Keywords with a centrality greater than 0.1 (marked by a purple outer ring in the graph) are considered highly influential. In the context of ocular drug delivery systems, keywords, such as controlled release (0.19), cell (0.14), dry eye (0.13), mechanism (0.12), expression (0.12), and ocular drug delivery (0.1) exhibit high centrality, making them key nodes in this research field.

### Keyword cluster analysis and timeline graph

**Figure 8.**
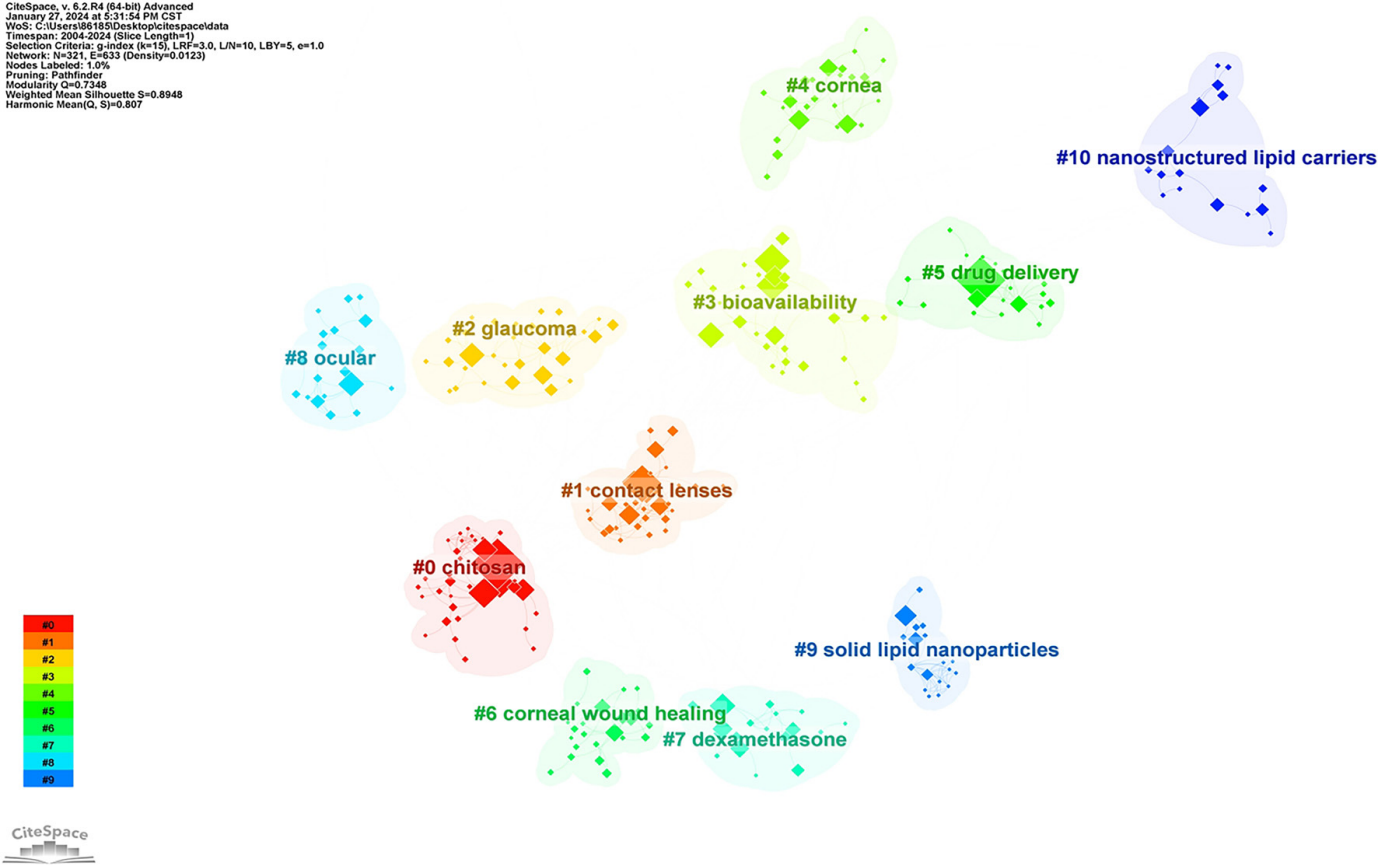
The co-occurrence analysis of the keyword cluster.

**Figure 9.**
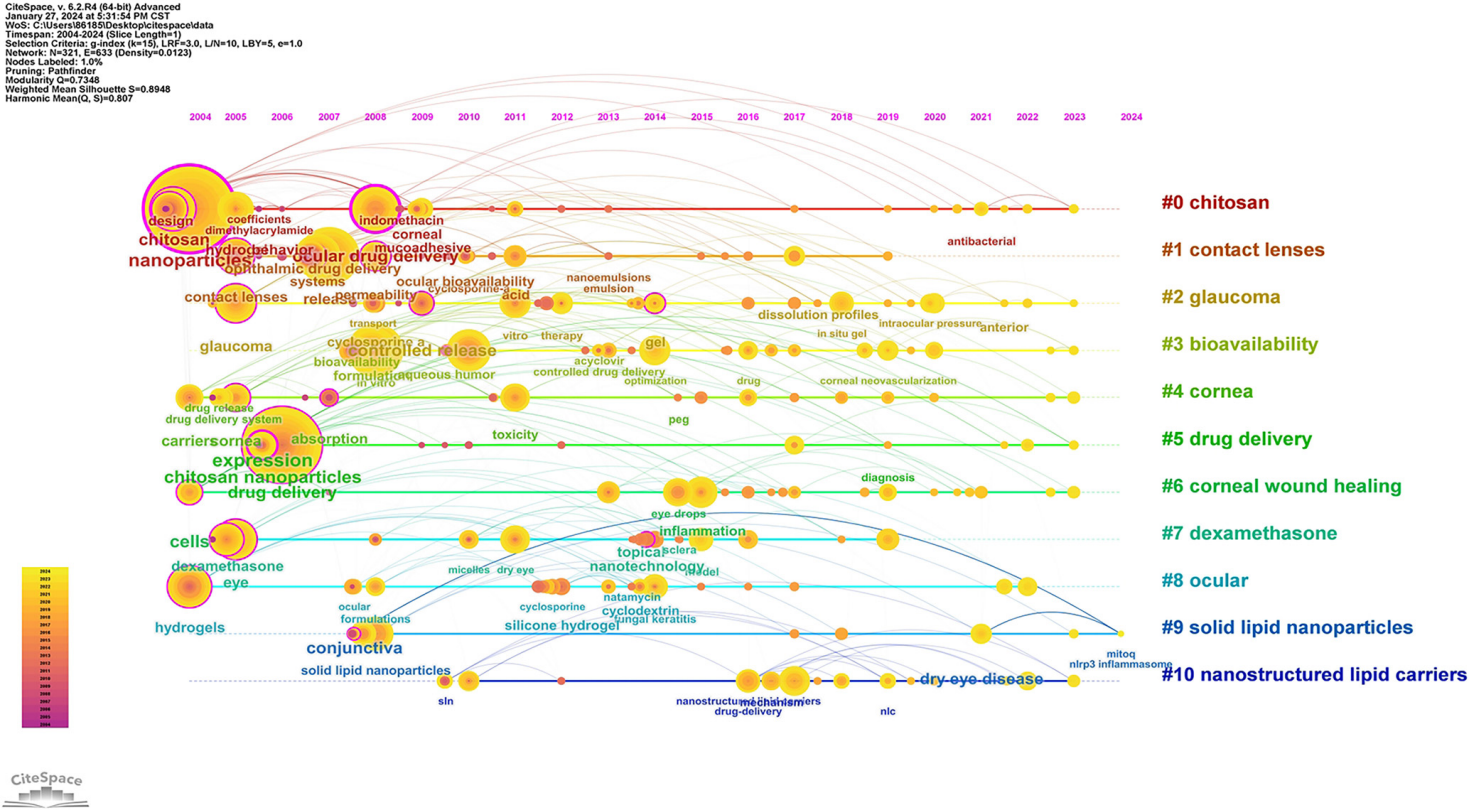
The co-occurrence analysis of the keyword timeline graph.

The keyword clustering map provides insight into the knowledge structure of research hotspots related to nanomediated ocular drug delivery systems and helps analyze the distribution of research topics. By automatically clustering 321 keywords, a cluster network display was generated, categorizing keywords in this field into 14 cluster modules, as shown in Figure 8. The modularity index (*Q*) was 0.7348 (*Q* values range from 0 to 1, with values above 0.3 indicating a significant network modular structure), while the silhouette index (S) was 0.8948 (S values closer to 1 indicate higher network homogeneity, with values above 0.5 suggesting a well-formed clustering structure). These indices confirm that the clustering is effective and that each cluster exhibits high internal homogeneity. An analysis of the clustering results reveals that research in ocular drug delivery systems over the past 20 years has focused on 11 key topics: #0 chitosan, #1 contact lenses, #2 glaucoma, #3 bioavailability, #4 cornea, #5 drug delivery, #6 corneal wound healing, #7 dexamethasone, #8 ocular, #9 solid LNPs, and #10 nanostructured lipid carriers. Additionally, a keyword timeline graph was generated using CiteSpace software to examine the development trends of various research topics related to nanomediated ocular drug delivery systems from 2004 to 2023 (Figure 9). This visualization highlights which research themes have gained traction in recent years and which ones are gradually declining in interest. Such insights can help researchers identify future directions and potential opportunities in this evolving field.

(1) Four research topics—#0 chitosan, #4 cornea, #8 ocular, and #6 corneal wound healing—emerged around 2004 and have remained active, making them some of the longest-standing research areas in the field. Early high-frequency keywords, such as nanoparticles, drug delivery, and ocular drug were commonly associated with these clusters. In recent years, new keywords like in situ gel, polymeric micelles, and antioxidant have also emerged, indicating that these topics continue to receive sustained research interest. (2) Three other topics—#1 contact lenses, #2 glaucoma, and #7 dexamethasone—appeared earlier, with key associated terms including release, permeability, hydrogels, and eye. Among them, #1 contact lenses and #7 dexamethasone saw a decline in research activity around 2020, while interest in glaucoma has persisted. (3) Three additional research clusters—#3 bioavailability, #9 solid LNPs, and #10 nanostructured lipid carriers—formed relatively late but continue to attract widespread attention. Notably, the #10 nanostructured lipid carriers cluster has seen the emergence of numerous new keywords since 2016, such as stability and nanostructured lipid carriers. A deeper analysis of the decline in chitosan-based systematic studies suggests possible contributing factors, including technical bottlenecks, cost issues, and application limitations. Meanwhile, the growing interest in LNP research may be driven by their unique physical and chemical properties, strong biocompatibility and biodegradability, and promising potential for ocular disease treatment.

## Discussion

In this study, an extensive body of literature on non-invasive nanoparticle drug delivery systems for ophthalmic disease treatment was collected through a comprehensive search and screening process. The selected literature was systematically analyzed and organized using bibliometric methods, including keyword co-occurrence and cluster analysis. The findings highlight the significant advantages and developmental potential of non-invasive nanoparticle drug delivery systems in ophthalmic treatment. The ocular surface, being in direct contact with the external environment, serves as a crucial site for non-invasive drug delivery. Transocular surface drug delivery systems, known for their efficiency and non-invasive nature, hold great promise in ophthalmic pharmaceuticals. In recent years, extensive research on ocular nanomediated drug delivery has been conducted across various laboratories, covering a wide range of diseases and applications. This growing body of work underscores the importance of this field for further exploration. Bibliometric analysis offers a quantitative approach to reviewing and evaluating existing literature in a specific domain [11, 20–22]. With the aid of modern computational tools, the results can be effectively visualized through clear and concise knowledge graphs. By examining publication volume, citation frequency, key publishers, leading journals, and major research themes in transocular surface drug delivery, emerging trends and research hotspots can be readily identified.

Although nanotechnology holds great promise for drug delivery, patient compliance and comfort remain key factors influencing treatment outcomes. This is especially important for drug delivery systems that require long-term wear or frequent use, such as nanoparticle-loaded contact lenses. Since tolerance to contact lens wear varies among individuals, some patients may discontinue use due to discomfort or allergic reactions. Therefore, optimizing the material, design, and fit of these lenses is essential to enhance comfort, improve patient acceptance, and ultimately boost compliance.

An analysis of publications in this domain reveals a steady annual increase in research output, indicating sustained interest among researchers. A review of 695 articles published between 2004 and 2023, spanning 58 countries/regions, 916 institutions, and 3405 authors, demonstrates the global engagement in this field. The co-occurrence network graph of institutions with a publication volume of 10 or more highlights that universities serve as the primary platforms for research on transocular surface nanomediated drug delivery systems. Furthermore, extensive inter-institutional collaboration is evident, forming a significant cooperative network. This suggests that researchers are not limited to small-group studies but actively collaborate across multiple institutions, advancing research through resource sharing. China leads in publication count in this field (*n* ═ 220), though Spain and the United States exhibit higher average citations per article. This aligns with the significant contributions of the most prolific authors—Carmen Álvarez-Lorenzo and Anuj Chauhan—and their respective institutions, the University of Santiago de Compostela and the University of Florida. Chauhan’s research team focuses on enhancing the efficiency and compliance of ophthalmic drug delivery, pioneering novel nanostructured soft contact lenses capable of sustained drug release over days to weeks, significantly reducing drug loss to systemic circulation. Their research targets various ophthalmic diseases, including glaucoma, dry eye syndrome, chemical burns, and allergies. Recently, they have begun exploring drug delivery to the posterior segment of the eye—traditionally treated with intraocular injections—investigating the feasibility of using contact lenses for this purpose. Carmen Álvarez-Lorenzo, a Spanish pharmacist and researcher, primarily focuses on the design and synthesis of polymer materials for drug release and nano drug delivery. Her work in ocular drug release aims to develop innovative systems that enhance drug efficacy and reduce dosing frequency. Notable achievements include polymer-based ophthalmic controlled-release gels, nanoscale ophthalmic controlled-release systems, and temperature-sensitive controlled-release systems. The International Journal of Pharmaceutics, with an impact factor of 5.8, is a leading academic journal in the pharmacy field, publishing original research on pharmaceutical formulations, controlled release systems, nanotechnology, biopharmaceutics, and drug delivery. It has contributed significantly to research in this domain, with 57 published articles. Notably, the journal with the highest average citations per article is the Journal of Controlled Release, which has a current impact factor of 11.467 and is recognized as one of the most influential journals in pharmacy, biomaterials, and drug delivery. Covering a wide range of drug release topics—including system design, release mechanisms, and drug transportation—it serves as a high-quality academic platform for researchers to communicate and share findings, playing a pivotal role in advancing progress in this field.

Through co-occurrence analysis, cluster analysis, and timeline diagram analysis of keywords, it is evident that research on nanoparticle drug delivery systems for the ocular surface has evolved over the past 20 years. In the early years, studies primarily focused on chitosan, whereas more recent research has shifted toward cationic liposomes. This transition suggests a gradual change in the preferred nanomaterials for ocular drug delivery, moving from chitosan-based systems to cationic liposomes. Chitosan, one of the most widely studied raw materials in nanomedicine, has been shown to exert antiviral and antibacterial effects when applied to the ocular surface. Chitosan-based nanoparticles have also been used to deliver cyclosporine, facilitating its immunomodulatory functions [23–25]. In recent years, advancements in chitosan-based nanomedicines have led to the development of collagen/chitosan microspheres for corneal epithelial regeneration [26] and a multifunctional hybrid hydrogel composed of silk fibroin and chitosan, which enables sustained drug release and has proven effective in treating fungal keratitis [27]. More recently, particularly in the wake of the COVID-19 pandemic, LNP-based mRNA vaccines have received FDA approval for clinical use, significantly driving research and applications in related fields. Several influential studies on LNPs have highlighted their long-term therapeutic potential. For instance, Baran-Rachwalska et al. [28] demonstrated that LNP-loaded siRNAs can efficiently target the corneal epithelium, offering new insights into treating corneal and anterior segment diseases while also providing a controlled method for siRNA-based interventions. Additionally, another study successfully employed LNP-mediated CRISPR/Cas9 genome editing for corneal gene modifications in mice, presenting a promising strategy for addressing corneal genetic disorders [29]. Given the existing use of LNP-loaded biologic drugs for clinical disease management, their potential for non-invasive ocular drug delivery holds significant translational value for future treatments. Nanoparticle-based formulations offer several unique advantages in ocular drug delivery, including small particle size, high penetration capacity, and controlled drug release. The mechanism of nanoparticle-mediated ocular drug delivery involves several key steps. First, nanoparticles must infiltrate and penetrate ocular tissues while overcoming barrier effects, such as the tight junctions of the corneal epithelium and endothelial cells. Next, they interact with ocular cells through adsorption, endocytosis, and translocation. Finally, the drug is released and exerts its therapeutic effects within the target cells. Compared to traditional ocular drug delivery methods (e.g., eye drops, ointments, and tablets), nanoparticle formulations address several limitations, such as short drug retention time, poor penetration, low patient compliance, and potential side effects from frequent administration. By leveraging nanotechnology, these advanced delivery systems enhance corneal penetration and prolong drug retention on the ocular surface, ultimately improving bioavailability and therapeutic efficacy.

In contrast to nanoparticle drug delivery research in other organs (e.g., skin, lungs, intestines), research on ocular surfaces also focuses on improving drug bioavailability, stability, and targeting. However, the unique anatomy and physiological properties of the ocular surface—such as rapid tear drainage and corneal barriers—have led to research priorities that specifically address these challenges. For example, corneal wound healing and dry eye treatment are key areas of focus in ocular drug delivery, whereas they may not be primary concerns in drug delivery studies for other organs. Earlier studies extensively explored the use of contact lenses as carriers for nanomedicine delivery. One study demonstrated that molecular blotting techniques significantly increased the timolol loading capacity of contact lenses, enabling prolonged drug release [30]. Additionally, researchers have developed an effective and long-lasting vancomycin delivery system using nanoparticle-containing contact lenses, offering a novel approach for treating infectious eye diseases [31]. Furthermore, a new type of polyacrylamide semi-interpenetrating network hydrogel, constructed with quaternized chitosan and tannic acid, has led to the development of antibacterial and antioxidant contact lenses. This advancement enables the synergistic treatment of infections and oxidative stress-related damage [32]. Another promising technology that has gained attention in ocular drug delivery is microneedles (MNs), which hold significant academic and clinical value. MNs allow for painless and efficient drug delivery through local tissues, making them particularly useful for treating eye diseases [33]. A 2007 study using a rabbit model demonstrated that fluorescein sodium and pilocarpine [34] could be effectively delivered via MNs. Additionally, corneal MNs have shown potential for intracorneal drug delivery, and advancements such as dissolvable needles and detachable single MN technologies offer new possibilities for localized treatment [35, 36]. Beyond direct drug delivery, recent studies have explored the use of MNs to administer iron-based riboflavin nanoparticles and riboflavin itself, utilizing photothermal and cross-linking effects to treat bacterial keratitis and facilitate corneal cross-linking [37, 38]. These findings underscore the need for further research into nanomaterial-based drugs with innovative carriers, while also promoting the clinical translation of emerging drug delivery technologies. For instance, non-invasive nanoparticle drug delivery systems can be leveraged for treating glaucoma—an eye disease characterized by optic nerve damage, often due to increased intraocular pressure. Prolonged high intraocular pressure can lead to the gradual degeneration of optic nerve fibers, ultimately resulting in vision loss. Non-invasive nanoparticle drug delivery systems offer a means to administer medications through the ocular surface (e.g., the conjunctival sac), utilizing the permeability and targeting properties of nanoparticles to directly reach ocular tissues. This approach can help lower intraocular pressure and minimize optic nerve damage. Potential drug candidates include beta-blockers, carbonic anhydrase inhibitors, and other therapeutics [39]. Similarly, nanoparticle drug delivery systems hold promise for treating age-related macular degeneration (AMD), one of the leading causes of vision loss in the elderly. AMD involves the degeneration of the macular region in the central retina. Nanoparticle-based systems can facilitate the ocular surface delivery of anti-vascular endothelial growth factor (VEGF) drugs and other therapeutics, inhibiting neovascularization and slowing disease progression [40]. Looking ahead, the future of non-invasive nanoparticle drug delivery systems will involve the development of novel nanomaterials with improved biocompatibility, stability, and targeting capabilities. These advancements aim to enhance the efficiency of ocular drug delivery and improve therapeutic outcomes. Additionally, intelligent drug delivery systems capable of responding to changes in the ocular microenvironment (e.g., pH and temperature) may enable precise drug release and personalized treatment strategies.

Based on the results of this study, we recommend that researchers further explore novel carrier materials for nanoparticles in ocular drug delivery to enhance drug stability and bioavailability. For clinicians, we emphasize the importance of closely monitoring patients’ ocular reactions and potential toxicity concerns when applying nanomedicines in clinical practice. Additionally, we encourage our industry partners to carefully consider cost-effectiveness and market demand when developing nanomedicine products to ensure their sustainability and competitiveness. In conclusion, this study conducted a bibliometric analysis using VOSviewer (version 1.6.20) and CiteSpace (version 6.1) to examine literature on noninvasive nanoparticle drug delivery systems for the transocular surface over the past twenty years. The analysis considered factors, such as publication volume, citation frequency, publishing institutions, journals, and research topics, offering a comprehensive understanding of the field. The novelty of this study lies in its interdisciplinary approach, integrating knowledge from biomaterials science, pharmaceutical formulation technology, nanotechnology, and ophthalmic medicine. By analyzing research hotspots and trends, this study highlights potential future research directions, including the exploration of novel nanomaterials, optimization of drug release systems, and enhancement of drug bioavailability. These insights contribute to the ongoing advancement of noninvasive transocular drug delivery systems. However, this study has certain limitations. The analysis relied solely on data from the WoSCC, which may introduce a bias toward English-language publications and potentially overlook significant research in other languages. Additionally, the formatting requirements of the visualization software may have excluded some data sources. Citation metrics can fluctuate over time, impacting both results and their interpretation. Furthermore, while bibliometrics provides valuable quantitative insights, it does not assess the qualitative aspects of research content. These limitations should be considered when interpreting and applying the study’s findings.

## Conclusion

This study systematically summarizes the current research status and trends in non-invasive ocular surface nanoparticle drug delivery systems using bibliometric analysis. The findings indicate that this field has grown rapidly in recent years, particularly in enhancing drug solubility, stability, corneal penetration, and prolonging drug residence time on the ocular surface—demonstrating significant advantages. Countries, such as China, the United States, India, and Spain have made notable contributions, forming a core group of research leaders. Keyword co-occurrence analysis identified key research themes, including controlled release, cellular interactions, dry eye, mechanisms, gene expression, and ocular drug delivery. Looking ahead, research in non-invasive ocular surface nanoparticle drug delivery will continue exploring novel nanomaterials to improve drug biocompatibility, stability, and targeting. Additionally, optimizing drug release systems and enhancing bioavailability will remain key focus areas. With advancements in smart technologies, the development of intelligent drug delivery systems capable of responding to changes in the ocular microenvironment is expected to become a major research direction. These innovations will drive the continued progress of ocular drug delivery technology, offering more effective and safer treatment options for patients with ocular diseases.
